# Supplementary material for: Axonal Organelle Buildup from Loss of AP-4 Complex Function Causes Exacerbation of Amyloid Plaque Pathology and Gliosis in Alzheimer's Disease Mouse Model
Source: eNeuro. 2024 Dec 19;11(12):ENEURO.0445-24.2024. doi: 10.1523/ENEURO.0445-24.2024 (PMC11775439; doi:10.1523/ENEURO.0445-24.2024)
Supplement: Table 1-1 — Antibody Summary. Download Table 1-1, DOCX file. [file eneuro-11-ENEURO.0445-24.2024-s004.docx]

**Table 1. Antibody Summary**

| Antibody | Source | Catalog number | Dilution |
| --- | --- | --- | --- |
| Amyloid-β | Cell Signaling Technology | 2454 | 1:500 |
| ARL8B | Invitrogen | PA5-98885 | 1:200 |
| BACE1  Cathepsin B | Cell Signaling Technology  R&D systems | D10E5  AF965 | 1:100  1:400 |
| Iba-1 | Wako | 019-19741 | 1:250 |
| LAMP1 | DSHB | 1D4B | 1:500 |
| mLegumain | R&D systems | AF2058 | 1:100 |
| mPGRN | R&D systems | AF2557 | 1:200 |
| RagC | Cell Signaling Technology | 9480S | 1:200 |
